# Supplementary material for: Contribution of markers of adiposopathy and adipose cell size in predicting insulin resistance in women of varying age and adiposity
Source: Adipocyte. 2022 Apr 18;11(1):175–89. doi: 10.1080/21623945.2022.2059902 (PMC9037496; doi:10.1080/21623945.2022.2059902)
Supplement: Supplemental Material [file KADI_A_2059902_SM8367.zip › supplementary/Figures Tremblay et alrevised without marks 2022 03 03.docx]

**Figure 1.**

**Figure 2.**

**Figure 3.**

**A**

**B**

| **A/L ratio tertile (mean ± SD; range)** | **BMI (kg/m^2^)** | **Body fat**  **(%)** | **Total fat area (cm^2^)** | **SCABD fat area (cm^2^)** | **Visceral fat area (cm^2^)** | **SCABD fat cell size (μm)** | **OME fat**  **cell size (μm)** |
| --- | --- | --- | --- | --- | --- | --- | --- |
| **Low** | 31.7 ± 4.8^a^  (24.3-41.3) | 40.4 ± 3.7^a^  (34.5-47.5) | 596 ± 167^a^  (370-991) | 462 ± 130^a^  (272-758) | 133 ± 46^a^  (86-232) | 109.9 ± 7.8^a^  (41.7-178.8) | 92.8 ± 10.2^a^  (35.2-214.0) |
| **Mid** | 27.5 ± 3.5^b^  (19.1-32.9) | 36.1 ± 4.4^b^  (24.2-41.1) | 444 ± 122^b^  (208-637) | 339 ± 102^b^  (170-539) | 105 ± 34^a^  (37-154) | 99.1 ± 10.6^b^  (31.1-199.4) | 84.1 ± 15.4^a^  (27.6-247.0) |
| **High** | 22.8 ± 2.7^c^  (17.2-29.1) | 29.4 ± 4.9^c^ (19.6-38.4) | 263 ± 85.5^c^  (128-422) | 206 ± 68.6^c^  (94-315) | 59 ± 22.0^b^  (33-131) | 88.2 ± 10.9^c^  (36.9-173.1) | 69.3 ± 4.2^b^  (24.3-156.1) |

**Figure 4.**

**A**

| Tertile cut-offs | Low | Mid | High |
| --- | --- | --- | --- |
| A/L ratio | ˂ 0.25 | 0.25 ≤ x ≤ 0.90 | ˃0.90 |
| SCABD adipose cell size (µm) | ˂ 91.0 | 91.0 ≤ x ≤ 105.5 | ˃105.5 |

**B**

| Tertile cut-offs | Low | Mid | High |
| --- | --- | --- | --- |
| A/L ratio | ˂ 0.25 | 0.25 ≤ x ≤ 0.90 | ˃0.90 |
| OME adipose  cell size (µm) | ˂ 71.0 | 71.0 ≤ x ≤ 90.0 | ˃90.0 |
