# Supplementary material for: Contribution of markers of adiposopathy and adipose cell size in predicting insulin resistance in women of varying age and adiposity
Source: Adipocyte. 2022 Apr 18;11(1):175–89. doi: 10.1080/21623945.2022.2059902 (PMC9037496; doi:10.1080/21623945.2022.2059902)
Supplement: Supplemental Material [file KADI_A_2059902_SM8367.zip › supplementary/Tables Tremblay et al revised without marks 2022 03 04.docx]

**Table 1.** Characteristics of the women studied.

**Table 2.** Spearman correlations between several AT dysfunction markers and patients’ lipid-lipoprotein profile.

**Tables 3.** Multiple regression models of predictors of glucose homeostasis.

**A.** Model including AT dysfunction markers without adipose cell size

**B.** Model including AT dysfunction markers with adipose cell size

**C**. Model including leptin levels

**Table 1.**

|  | n | Mean (SD) | Range (min - max) |
| --- | --- | --- | --- |
| Age (years) | 67 | 47 (5) | 40 - 62 |
| *Anthropometry and body fatness* | | | |
| Body weight (kg) | 65 | 70.6 (14.8) | 48.5 - 110.5 |
| Body mass index (kg/m²) | 65 | 27.2 (5.0) | 17.2 - 41.3 |
| Body fat mass (kg) | 65 | 25.5 (9.0) | 10.0 - 50.8 |
| Body fat percentage (%) | 65 | 35.1 (6.0) | 32.5 - 63.0 |
| Lean body mass (kg) | 65 | 43.1 (6.6) | 19.6 - 47.5 |
| *Abdominal adipose tissue areas (cm²)* | | | |
| Total | 63 | 424 (180) | 128 - 991 |
| Subcutaneous | 63 | 328 (141) | 94 - 759 |
| Visceral | 63 | 97 (46) | 34 - 233 |
| *Adipose cell size (µm)* | | | |
| Subcutaneous abdominal | 63 | 98.5 (12.9) | 66.8 - 122.7 |
| Omental | 59 | 80.8 (16.2) | 51.7 - 118.7 |
| *Lipid-lipoprotein profile* | | | |
| Cholesterol (mmol/L) | 62 | 4.81 (0.64) | 3.43 - 6.12 |
| LDL-cholesterol (mmol/L) | 61 | 2.71 (0.59) | 1.31 - 3.96 |
| HDL-cholesterol (mmol/L) | 62 | 1.49 (0.39) | 0.81 - 2.69 |
| Cholesterol/HDL-C | 62 | 3.43 (0.93) | 1.64 - 6.22 |
| Triacylglycerols (mmol/L) | 62 | 1.34 (0.74) | 0.51 - 4.69 |
| *Glucose homeostasis* | | | |
| Fasting glucose (mmol/L) | 63 | 5.6 (0.6) | 4.6 - 7.8 |
| Fasting insulin (pmol/L) | 63 | 11.4 (5.6) | 3.4 - 27.6 |
| HOMA-IR | 63 | 2.9 (1.7) | 0.8 - 8.8 |
| *Adipokines* | | | |
| Adiponectin (μg/mL) | 63 | 10.8 (5.8) | 0.7 - 28.6 |
| Leptin (ng/mL) | 61 | 26.4 (20.3) | 0.5 - 72.4 |
| Adiponectin/Leptin (10^-3^) | 60 | 2.7 (6.9) | 0.04 - 34.2 |
| *Macrophage infiltration markers* | | | |
| Subcutaneous abdominal | | | |
| CD68 mRNA | 51 | 1.26 (0.77) | 0.39 - 5.03 |
| CD11b mRNA | 52 | 0.96 (0.54) | 0.25 - 2.53 |
| CD11c mRNA | 52 | 1.72 (1.39) | 0.22 - 7.08 |
| Omental | | | |
| CD68 mRNA | 53 | 1.17 (0.53) | 0.46 - 3.13 |
| CD11b mRNA | 53 | 0.98 (0.54) | 0.30 - 2.54 |
| CD11c mRNA | 54 | 0.84 (0.68) *** | 0.19 - 2.92 |

SD, standard deviation. C, cholesterol; HDL, high-density lipoprotein; CD: cluster designation; LDL, low-density lipoprotein; HOMA-IR, HOmeostasic Model Assessment of Insulin Resistance. ***compared to subcutaneous abdominal at p<0.0005.

**Table 2.**

|  | Plasma  A/L ratio  (n = 55-58) | SCABD adipose  cell size  (n = 56-57) | OME adipose  cell size  (n = 54-56) | SCABD CD68  mRNA  (n = 47) | OME  CD68  mRNA  (n = 49) | SCABD CD11b mRNA  (n = 48) | OME CD11b mRNA  (n =49-50) | SCABD CD11c mRNA  (n = 48) | OME CD11c mRNA  (n = 50) |
| --- | --- | --- | --- | --- | --- | --- | --- | --- | --- |
| Cholesterol (mmol/L) | -0.13 | 0.13 | 0.16 | 0.24 | -0.06 | 0.08 | 0.17 | 0.15 | 0.10 |
| LDL-cholesterol (mmol/L) | -0.16 | 0.22 | 0.28* | 0.32* | 0.14 | 0.13 | 0.23 | 0.20 | 0.15 |
| HDL-cholesterol (mmol/L) | 0.38** | -0.48*** | -0.48******* | -0.28*** | -0.37* | -0.34* | -0.27 | -0.25 | -0.14 |
| Cholesterol/HDL-C | -0.43** | 0.52*** | 0.54*** | 0.31*** | 0.35* | 0.33* | 0.34* | 0.27 | 0.23 |
| Triacylglycerols (mmol/L) | -0.46*** | 0.46*** | 0.37** | 0.30* | 0.16 | 0.26 | 0.26 | 0.11 | 0.09 |

SCABD: subcutaneous abdominal; OME: omental. For other abbreviations, see legends to Table 1. ***p<0.0005, **p<0.005, *p<0.05.

**Tables 3.**

**A.**

| **SCABD** | | | | | **OME** | | | |
| --- | --- | --- | --- | --- | --- | --- | --- | --- |
| **HOMA index (IR)** | | | | | | | | |
|  | Model *R*^2^ = 0.38 | | |  | | Model *R*^2^ = 0.40 | | |
|  | β | Partial *R*^2^ | *p* |  |  | β | Partial *R*^2^ | *p* |
| A/L ratio | -0.428 | 0.32 | 0.0055 | A/L ratio | | -0.445 | 0.31 | 0.0021 |
| Triacylglycerols | 0.180 | 0.04 | NS | Triacylglycerols | | 0.315 | 0.05 | 0.0277 |
| CD68 | 0.168 | 0.02 | NS | CD68 | | -0.185 | 0.04 | NS |
| **Fasting insulin** | | | | | | | | |
|  | Model *R*^2^ = 0.36 | | |  | | Model *R*^2^ = 0.15 | | |
|  | β | Partial *R*^2^ | *p* |  |  | β | Partial *R*^2^ | *p* |
| A/L ratio | -0.492 | 0.33 | 0.0005 | A/L ratio | | -0.475 | 0.32 | 0.0011 |
| CD68 | 0.217 | 0.03 | NS | Triacylglycerols | | 0.282 | 0.05 | 0.0467 |
| Triacylglycerols | NE | - | - | CD68 | | -0.187 | 0.03 | NS |
|  | | | | | | | | |
| **Fasting glucose** | | | | | | | | |
|  | Model R^2^ = 0.14 | | |  | | Model *R*^2^ = 0.15 | | |
|  | β | Partial *R^2^* | p |  | | β | Partial *R*^2^ | *p* |
| Triacylglycerols | 0.374 | 0.14 | 0.0033 | Triacylglycerols | | 0.374 | 0.15 | 0.0033 |
| A/L ratio | NE | - | - | A/L ratio | | NE | - | - |
| CD68 | NE | - | - | CD68 | | NE | - | - |

For abbreviations, see legends to Tables 1 and 2; NE: not entered; NS: non-significant.

**B.**

| **SCABD** | | | | **OME** | | | | |
| --- | --- | --- | --- | --- | --- | --- | --- | --- |
| **HOMA index (IR)** | | | | | | | | |
|  | Model *R*^2^ = 0.45 | | | |  | Model *R*^2^ = 0.36 | | |
|  | β | Partial *R*^2^ | *p* | |  | β | Partial *R*^2^ | *p* |
| Adipose cell size | 0.552 | 0.43 | <0.0001 | | Adipose cell size | 0.391 | 0.30 | 0.0326 |
| CD68 | 0.139 | 0.02 | NS | | A/L ratio | -0.266 | 0.03 | NS |
| A/L ratio | NE | - | - | | CD68 | -0.154 | 0.03 | NS |
| **Fasting insulin** | | | | | | | | |
|  | Model *R*^2^ = 0.44 | | | |  | Model *R*^2^ = 0.37 | | |
|  | β | Partial *R*^2^ | *p* | |  | β | Partial *R*^2^ | *p* |
| Adipose cell size | 0.540 | 0.42 | 0.0002 | | Adipose cell size | 0.397 | 0.31 | 0.0288 |
| CD68 | 0.130 | 0.02 | NS | | A/L ratio | -0.272 | 0.04 | NS |
| A/L ratio | NE | - | - | | CD68 | -0.157 | 0.02 | NS |
|  | | | | | | | | |
| **Fasting glucose** | | | | | | | | |
|  | Model *R*^2^ = 0.23 | | | |  | Model *R*^2^ = 0.09 | | |
|  | β | Partial *R*^2^ | *p* | |  | β | Partial *R*^2^ | *p* |
| Adipose cell size | 0.414 | 0.23 | 0.0014 | | Adipose cell size | 0.316 | 0.09 | 0.0187 |
| CD68 | NE | - | - | | CD68 | NE | - | - |
| A/L ratio | NE | - | - | | A/L ratio | NE | - | - |

For abbreviations, see legends to Tables 1 and 2; NE: not entered; NS: non-significant.

**C.**

| **SCABD** | | | | **OME** | | | | |
| --- | --- | --- | --- | --- | --- | --- | --- | --- |
| **HOMA index (IR)** | | | | | | | | |
|  | Model *R*^2^ = 0.55 | | | |  | Model *R*^2^ = 0.45 | | |
|  | β | Partial *R*^2^ | *p* | |  | β | Partial *R*^2^ | *p* |
| Leptin | 0.427 | 0.46 | 0.0069 | | Leptin | 0.490 | 0.40 | 0.0041 |
| Adipose cell size | 0.305 | 0.07 | NS | | Adipose cell size | 0.236 | 0.03 | NS |
| CD68 | 0.157 | 0.02 | NS | | CD68 | -0.152 | 0.02 | NS |
| **Fasting insulin** | | | | | | | | |
|  | Model *R*^2^ = 0.53 | | | |  | Model *R*^2^ = 0.45 | | |
|  | β | Partial *R*^2^ | *p* | |  | β | Partial *R*^2^ | *p* |
| Leptin | 0.431 | 0.45 | 0.0072 | | Leptin | 0.478 | 0.40 | 0.0048 |
| Adipose cell size | 0.293 | 0.06 | NS | | Adipose cell size | 0.255 | 0.03 |  |
| CD68 | 0.147 | 0.02 | NS | | CD68 | -0.154 | 0.02 |  |
|  | | | | | | | | |
| **Fasting glucose** | | | | | | | | |
|  | Model *R*^2^ = 0.26 | | | |  | Model *R*^2^ = 0.17 | | |
|  | β | Partial *R*^2^ | *p* | |  | β | Partial *R*^2^ | *p* |
| Adipose cell size | 0.233 | 0.23 | NS | | Leptin | 0.321 | 0.13 | 0.0117 |
| Leptin | 0.270 | 0.03 | NS | | Adipose cell size | NE | - | - |
| CD68 | NE | - | - | | CD68 | NE | - | - |

For abbreviations, see legends to Tables 1 and 2; NE: not entered; NS: non-significant.
